# How to measure the effectiveness of recovery community centers: insights gained from a nationwide survey of directors of RCCs

**DOI:** 10.3389/fpubh.2025.1532812

**Published:** 2025-07-23

**Authors:** Bettina B. Hoeppner, Alivia C. Williamson, Hazel Simpson, Diadora DeCristofaro, Catherine Weerts, Marion J. Riggs, Allison Futter, Amy A. Mericle, Philip X. Rutherford, Lauren A. Hoffman, Vinod Rao, Patty McCarthy, Julia Ojeda, Susanne S. Hoeppner

**Affiliations:** ^1^Department of Psychiatry, Massachusetts General Hospital, Boston, MA, United States; ^2^Harvard Medical School, Boston, MA, United States; ^3^Department of Neurology, Harvard Medical School, Boston, MA, United States; ^4^Alcohol Research Group, Public Health Institute, Emeryville, CA, United States; ^5^Black Faces Black Voices, Rochester, MN, United States; ^6^Faces & Voices of Recovery, Washington, DC, United States; ^7^Recovery Inspired Opportunities, Boston, MA, United States

**Keywords:** recovery community centers, peer recovery support services, substance use disorder, recovery, addiction, definition of recovery, measurement of outcomes, measuring peer impact

## Abstract

**Objective:**

Recovery community centers (RCCs) are a rapidly growing source of support for many Americans seeking or in recovery from substance use disorder. Research that examines the effectiveness of RCCs is critically needed. Determining how the “effectiveness” of RCCs ought to be measured, however, is challenging, because RCCs seek to confer benefits on multiple levels and because recovery is a multi-faceted construct. RCC directors are uniquely suited to provide insight into what outcome measures may be viable to use to capture the impact of RCCs on their participants.

**Methods:**

As part of a nationwide survey of RCCs directors (*n* = 122/198, 62% response rate), we presented seven measures to RCC directors: process measures (Client Satisfaction Questionnaire (CSQ-8); a list of ways in which RCCs confer benefit) and longer term outcome measures (Brief Assessment of Recovery Capital (BARC-10); EUROHIS-QOL; Substance Use Recovery Evaluator (SURE); PERMA Profiler; a list of life goals). We then asked RCC directors if they expected that RCC participants would show progress over time on these measures (yes/no), if the measure would be useful to demonstrate the impact of their RCC (yes/no), and which measure they felt was the best measure of the positive impact RCCs can make on RCC participants.

**Results:**

All measures had considerable buy-in from RCC directors: 87% or more of RCC directors said RCC participants would show progress on each measure, and 72% or more said that each measure would be useful to demonstrate the impact of their RCC. Most frequently, RCC directors endorsed as useful the list of ways in which RCCs confer benefit, the list of life goals, and the BARC-10 (≥95% of RCC directors each). RCC directors were split on which measure would be the best measure, with no single measure exceeding 26% of RCC directors rating it as the best of the seven presented scales.

**Conclusion:**

Several existing scales resonate with RCC directors, yet little consensus regarding a single primary outcome variable exists. Close collaboration with RCCs is needed to ensure that research on the effectiveness of RCCs is congruent with how RCCs function and seek to confer benefit.

## Introduction

Recovery community centers (RCCs) are emerging as an important third component of the recovery support landscape that, until recently, was comprised solely of professional treatment and mutual-help organizations (1). RCCs are brick and mortar places open to the community that serve as a central recovery hub by providing a variety of support services for people seeking or in recovery (2). Under the guiding principle of “all pathways are welcome”, RCCs generally host a myriad of mutual help groups (e.g., “all recovery” meetings, 12-step groups, mental health support groups), provide recovery coaching, access to technology (e.g., use of computers), and assistance with basic needs and social services. Many RCCs also offer space and guidance for engaging in health behaviors (e.g., nutrition programs, yoga). Beyond services, RCCs provide a space and foster a community for their participants to engage in substance-free recreational activities (e.g., birthday parties, board-gaming nights), often including opportunities for expressive art (e.g., arts/crafts groups, music) (2). RCCs are staffed by a small number of paid staff, and receive support from volunteers to deliver their services and host events (2). In these diverse ways, RCCs provide an opportunity for those seeking recovery or early in recovery to receive support, and an opportunity to “give back” to the community for those who are further along the path of recovery. On the community level, RCCs put a visible, de-stigmatizing face on recovery, and serve as a convenient, easily-accessible base of operations for the local recovery community (3). Thus, RCCs address multiple needs by providing knowledge, insight, services, and a recovery-supportive social environment.

RCCs are a rapidly growing source of community recovery support. A nationally representative survey of US adults conducted in 2016 showed that 6.2% of adults who have had a substance use problem but no longer do have used a recovery community center (4). Recent years have witnessed increased investment in RCCs. In Massachusetts, for example, the number of RCCs has increased more than 7-fold over 20 years, from five state-funded RCCs operating in 2008 to 39 operating in 2024. Nationwide, data from our survey study suggest that the number of RCCs has more than doubled in the 5 years prior to 2022 (2). Since 2022, RCCs are highlighted as a key resource in the U.S. National Drug Control Strategy (5).

Critically needed is research that examines the effectiveness of RCCs (6, 7). By “effectiveness”, we are referring to “the impact of an intervention on important individual outcomes, including potential negative effects, and broader impact including quality of life and economic outcomes; and variability across subgroups (generalizability or heterogeneity of effects)”, which is the definition of effectiveness used in the RE-AIM (Reach, Effectiveness, Adoption, Implementation, and Maintenance) framework (8). To date, no randomized clinical trial (RCT) has been conducted that provides gold-standard evidence of the effectiveness of RCCs. This type of evidence is needed to further support investment in RCCs, and to stimulate referral to RCCs. Such research can also shed light on best practices to guide new RCCs. Conducting effectiveness research on RCCs is challenging, however, for many reasons, chief among them the heterogeneity of approaches and services offered by RCCs, the different settings in which they operate, both in terms of their physical setting (e.g., urban, rural) and their surrounding socio-political climate (e.g., Northeast, Deep South), and the differences in funding streams they can utilize to operate (e.g., state-funded, philanthropy funded). Even before this complexity can be addressed, however, a more basic question arises: how do you define the “effectiveness” of an RCC? RCCs are designed to confer benefits on multiple levels. Providing support to individuals struggling with substance use disorder (SUD) is a centralizing focus, but certainly not the only one. Beyond the individual level, RCCs also are designed to confer benefits on the interpersonal, community, state and national level. They provide an opportunity to those further along the path to “give back” to the community, thereby supporting the growth of interpersonal and professional skills. RCCs impact their surrounding community, which through the myriad of outreach activities hosted by RCCs are prompted to think about recovery, and to re-think and question prejudices and stereotypes. Such stereotypes get challenged by experiencing people in recovery, and engaging with their life-stories; storytelling is an increasingly recognized mechanism for dismantling SUD stigma (9). On an even more macro-level scale, RCCs play a critical role in informing and impacting state and national policies through their collaboration with each other and their joint advocacy work. Thus, in considering the “effectiveness” of RCCs, it must be remembered that supporting individuals is only part of the mission of RCCs.

Even on the individual level, defining “effectiveness” of RCCs in supporting individuals struggling with SUD is complex. In SUD treatment settings, “success” has traditionally been defined as achieving abstinence (10, 11). The suitability of this narrow focus has been questioned for professionally delivered psychosocial treatments (12), and contrasts with the emergence of the concept of recovery (13), a concept which has established itself as the desired outcome for people with SUD. This emerging consensus is based, in part, on studies that have challenged the utility of focusing on abstinence as the key indicator of recovery. Recent large-scale studies have highlighted that (at least for alcohol) non-abstinent recovery is not only possible, but common (14, 15), leading to the argument that patterns of use are informative, but abstinence itself is of limited utility in defining success in recovery (16). Note also that for many persons who struggle with SUD or who engage in treatment, abstinence is not their goal (17). A focus on abstinence is certainly at odds with the vision and mission of RCCs, which are guided by the principles of recovery, a core part of which is that “recovery looks different for different people” (18).

In calling for research on the effectiveness of recovery support services, such as RCCs, the National Institute on Drug Abuse (NIDA) has offered the perspective that “recovery support services are thought to assist individuals with the recovery process” (as stated in RFA-DA-20-014). Fortunately, much progress has been made over the last decade in defining recovery. A consensus has emerged that recovery is a multi-faceted construct. NIDA defines it as: “a process of change through which people improve their health and wellness, live self-directed lives, and strive to reach their full potential.” The definition of recovery used by the Substance Abuse and Mental Health Services Administration (SAMHSA) (19), a federal agency within the U.S. Department of Health and Human Services that leads public health efforts to advance the behavioral health of the nation more broadly, is identical. Additionally, SAMHSA notes that there are “four major dimensions that support a life in recovery: health, home, purpose, and community.” (19) These four dimensions are encompassed by (1) making informed, healthy choices that support physical and emotional wellbeing, (2) having a stable and safe place to live, (3) engaging in meaningful daily activities, and having the independence and resources to participate in society, and (4) having relationships and social networks that provide support, friendship, love and hope. Thus, an overall conceptual consensus has been established that defines recovery as a multi-faceted construct encapsulating diverse outcomes.

RCCs provide support for all of these diverse outcomes—in a variety of ways and to varying degrees. In examining the effectiveness of RCCs, it is necessary to operationally define these multi-faceted outcomes, so as to be able to measure them. It is one thing to agree that recovery encompasses multiple, specific outcomes. It is quite another to agree on specific measures that validly capture the degree to which individuals are achieving these markers of recovery.

In an effort to inform future research efforts that seek to test the effectiveness of RCCs, we sought input from RCC directors on measures that could be used to capture the impact an RCC can have on its participants. RCC directors are key constituents in defining outcomes of recovery community centers, as they are instrumental in designing the services RCCs offer, and typically have lived experience of going through the process of recovery. Thus, RCC directors are experts on this specific intervention/service delivery mechanism (i.e., RCCs) and the goals it should achieve, which is why their input on selecting outcome measures for effectiveness research is of paramount importance. To this end, we conducted a nationwide survey of RCC directors. Previously, we reported on part 1 of the survey, in which we asked RCC directors to describe their RCC (2). Here, we report on part 2 of the survey, in which we presented RCC directors with specific outcome measures, and asked for their evaluation of them.

In designing this study, we were guided by reactions to the conclusions arrived at by an expert panel convened by the National Institute of Drug Abuse (NIDA) well over a decade ago to address addictions treatment outcome issues (20, 21). In reacting to those recommendations, del Boca and Darkes offered three guiding principles for future work: (1) the selection of outcome measures should reflect and inform evolving conceptualizations of recovery, (2) measures should, in addition to reliability and validity, demonstrate sensitivity to change, and (3) outcome measures should have clinical relevance (22). To this end, we selected, for the most part, scales of known robust psychometric properties that were in line with the evolving conceptualization of recovery. We then asked RCC directors to evaluate the “clinical” relevance of these selected measures, and their ability to demonstrate change over time. In selecting measures for feedback, we included both process measures (e.g., satisfaction with services) and downstream, longer-term outcomes (e.g., quality of life), which are conceptually distinct parts of complex interventions (23).

## Methods

### Participants

Participants were directors of RCCs or their delegates (e.g., center manager, associate director) located anywhere in the continental US. No central locator of RCCs exists; thus, RCCs were identified via smaller existing lists of RCCs, online searches, and networking, as described in our previous paper on this study (2). Our search identified 198 RCCs fitting the definition: “Brick and mortar places located within the heart of a community that serve as a central recovery hub by providing a variety of support services for people in or seeking recovery”. Of these, 122 (62%) RCC directors participated in our survey, and 114 completed part 2 of the survey, which pertained to providing feedback on outcome measures.

### Procedure

Study staff sent email invitations to RCC directors and followed up via phone on three or more days. The email invitations contained an RCC-specific survey link (only one survey could be completed per RCC), administered via the secure, web-based, HIPAA-compliant data capture system REDCap (24). In part 1 of the survey, RCC directors or their delegates answered multiple-choice questions describing their RCC, as described and reported on previously (2). In part 2, they were asked to review specific measures, and then answered questions about them. This transition in the survey was clearly demarked using the following text: “Thank you for completing Part 1 of this survey! […] In Part 2, we will now ask you about your opinion about potential measures that could be used to measure the impact of RCCs on the individuals and communities they serve.” In phrasing this introduction (and subsequent survey items), we deliberately used the word “impact” rather than “effectiveness”, as the word “effectiveness” has a very specific meaning in health behavior change research, which RCC directors may not be aware of. Thus, we chose the word “impact” for ease of understanding. Note that this word captures the main thrust of the definition of “effectiveness”, as defined in the RE-AIM framework, as effectiveness is “the impact of an intervention on important individual outcomes” (8). RCC directors were free to skip questions they did not want to answer.

### Measures

In the current paper, we included the basic descriptors (from survey part 1) specific to the *n* = 114 RCCs that participated in part 2 of the survey: information about RCC staffing, RCC footprints, RCC model of care, and estimated demographics of RCC participants. Of note, to estimate demographics of the participants of their RCC, RCC directors were asked to provide their “best guess to estimate the percent of people who fall into each category at your RCC”. For each variable (age, gender, ethnicity, race), RCC directors were provided multiple textboxes in which to indicate the percentage of their RCC participants who would be best described by this category. For example, for ethnicity, two textboxes were provided, which read “Hispanic/Latino” and “Not Hispanic/Latino”. Right underneath these two textboxes was a calculated field, labeled “Total (please make sure this equals 100%)”. This field provided the sum of the percentages RCC directors provided in the textboxes matching each category, so that they could easily see if their estimated percentages added up to 100%.

In part 2, the survey presented seven measures to RCC directors: two process measures capturing RCC participants' short-term appraisals of their experience with the RCC (i.e., satisfaction with services; RCC specific reflections) and RCC participants' downstream, longer-term outcomes (i.e., recovery capital, quality of life, recovery, flourishing, and achievement of life goals). We included both short-term appraisals and longer term outcomes because they are distinct and important parts of complex interventions (23). The reasons we selected each measure to include in this study is summarized in Table 1, and detailed for each measure in the measure-specific description below.

**Table 1:** Rationale for selecting outcome measures for evaluation by RCC directors.

**Type of Measure**	**Why we selected it**
**Process measures (i.e., is the RCC delivering support as intended)**	
Client Satisfaction Questionnaire (CSQ-8)	- widely used measure of client and patient satisfaction (cited 3,600+ times)
	- provides insight into how RCC participants experience RCC support
Ways in Which RCCs Confer Benefits	- designed to capture RCC specific experiences, thoughts, and feelings
	- developed by our team based on our knowledge of RCCs
	- guided by knowledge shared in monthly online seminar series on RCCs
**Outcome measures (i.e., is the RCC impacting recovery outcomes)**	
Brief Assessment of Recovery Capital (BARC-10)	- frequently used measure of recovery capital in recovery science
	- used by many RCCs via the Recovery Data Platform (RDP)
EUROHIS-QOL	- widely used measure of quality of life
	- used to calculate quality-adjusted life years (QALYs)
Substance Use Recovery Evaluator (SURE)	- iteratively created with people in recovery and SUD clinicians
	- assesses six dimensions of recovery; abstinence not required
	- encompasses all four dimensions of recovery highlighted by SAMHSA
	- relatively novel, but starting to be used in RCTs in SUD research
PERMA profiler	- shifts the focus from functioning to flourishing
	- widely used internationally to measure flourishing (cited 2,100+ times)
Life Goals	- designed to capture NIDA's theme on “regain health and social function”
	- developed by our team based on our knowledge of RCCs
	- congruent with NIDA's and SAMHSA's definition of recovery

**Figure 1:**
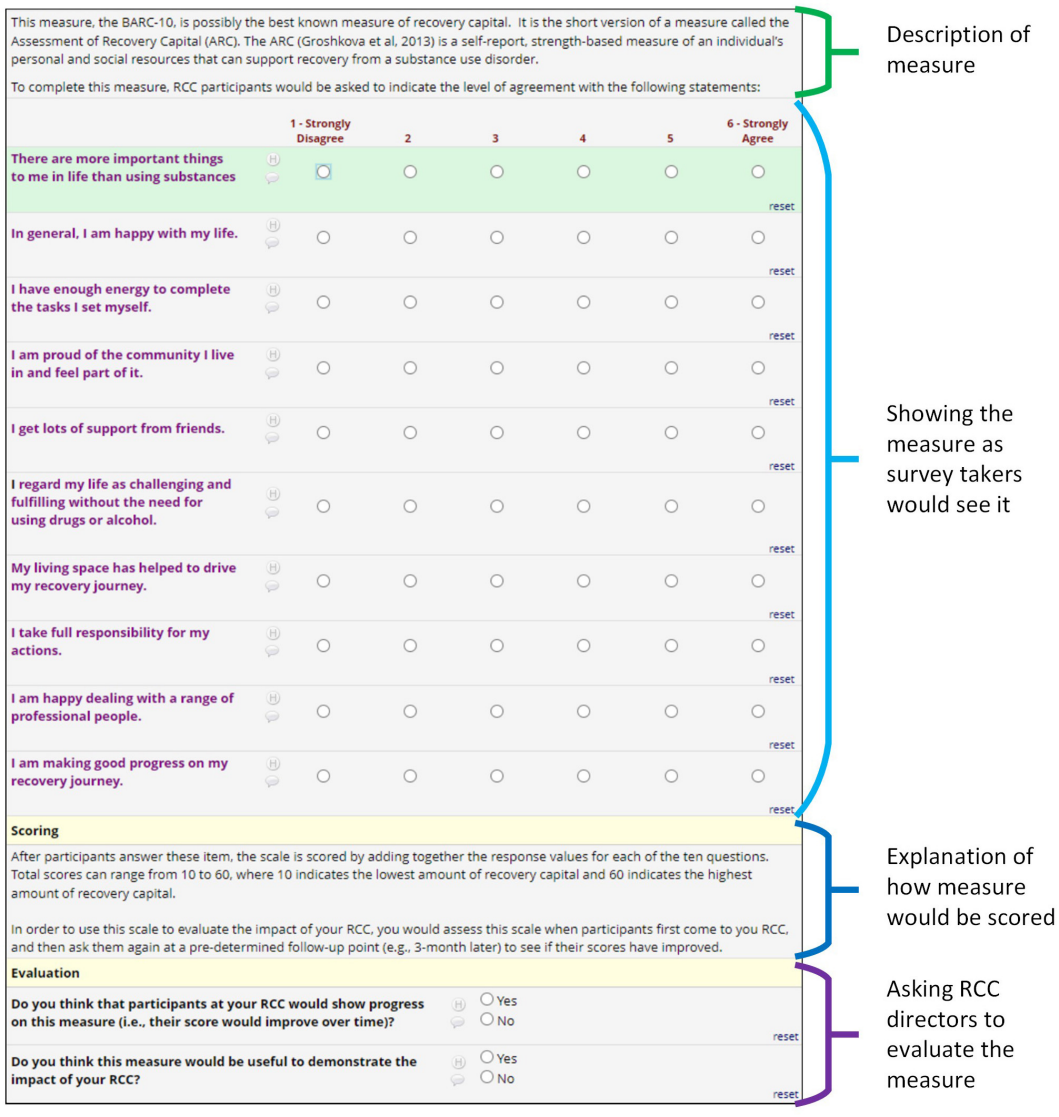
Screenshot of REDCap survey asking directors to provide feedback on selected measures.

For each outcome measure, the survey provided information about the measure before asking RCC directors to answer questions about it: (1) background information about the measure, including information about how and when RCC participants would be asked to provide data on this measure (e.g., once, baseline and follow-up); (2) the actual survey items as participants would see them, including their responses options; and (3) scoring information, which explained how scores would be calculated, and how this score would be interpreted. This process is illustrated in Figure 1, using one of the seven outcome measures as an example (please see Supplementary material #1 for screenshots of how we presented all measures in this survey). After reviewing this information, RCC directors were then asked to respond to two yes/no items: “Do you think that participants at your RCC would show progress on this measure?”; depending on the nature of the measure, parenthetical text either added “(i.e., their score would improve over time)” or “(i.e., that they would check off any of these boxes)”; and “Do you think this measure would be useful to demonstrate the impact of your RCC?”. If they indicated “no”, the survey asked them to provide additional thoughts in a textbox, using the prompt “please explain”.

After RCC directors rated each of the seven outcome measures, we asked “Now that you have taken a look at the 7 measures we thought are relevant to assessing the positive impact of RCCs, which one do you think is the best measure of the positive impact RCCs can make on people seeking or in recovery?” and “Which measure do you think is the worst measure of the positive impact RCC can make on people seeking or in recovery?”

In terms of short-term process measures (Table 1), we presented two measures:

Client Satisfaction Questionnaire (CSQ-8). The CSQ-8 consists of 8 items that ask participants to evaluate “the service they have received” using a 4-point Likert scale (25). Items include: “How would you rate the quality of service you received?” (response options ranging from “poor” to “excellent”), and “Did you get the kind of service you wanted?” (response options ranging from “no, definitely not” to “yes, definitely”). The CSQ-8 was first published in 1979, and is a well-known and widely used measure of client and patient satisfaction (cited 3,600+ times).

Ways in which RCCs Confer Benefits. To assess RCC-related experiences more directly, our team developed a measure that queries RCC participants about their thoughts and feelings regarding their interactions with their RCC. To this end, our team generated 17 items with the stem “The RCC I attend …” followed by statements about specific ways in which participants may have experienced a beneficial impact of their interactions with an RCC (e.g., “… gave me confidence to persist in my recovery”; “… was a safe haven in a moment of crisis”; “… gave me trusted advice whenever I needed it”, etc.). We generated these items based on our knowledge of how RCCs function (our authorship team includes recovery leaders involved in the accreditation of RCCs and a former state employee overseeing the implementation of RCCs), and as shared in the monthly seminar series our team leads on RCCs, which brings together key community groups interested in advancing the science on RCCs (e.g., RCC directors, RCC staff, RCC participants, RCC advocates, RCC funders, recovery scientists, SUD clinicians). Participants would rate these statements on a 6-point Likert scale to indicate their level of agreement with that statement (response options ranging from “strongly disagree” to “strongly agree”; please see Supplementary material #2). In our survey, we presented the items we had generated, and asked RCC directors to add items that should be added to this list.

In terms of longer-term outcome measures (Table 1), we presented five measures:

Brief Assessment of Recovery Capital (BARC-10). The BARC-10 (26) is a shortened version of the 50-item long Assessment of Recovery Capital (ARC) (27). It consists of ten items, rated on a 6-point Likert scale (ranging from “strongly disagree” to “strongly agree”), and includes items such as: “There are more important things to me in life than using substances” and “I get lots of support from friends”. It is well-known among scientists and RCC directors. Among scientists, the BARC-10 stands out as one of the most widely used measures of recovery capital (28), where recovery capital is defined as “…the sum of one's total resources that can be brought to bear in an effort to overcome alcohol and drug dependency” (29). Among RCC directors, it stands out as the most frequently used measure to capture recovery progress in their participants, assessed as part of their routine recovery support activities. Many RCCs use the Recovery Data Platform (RDP)™ to collect data akin to electronic health records, and to document recovery progress over time. The BARC-10 has been programmed into the RDP since 2018 as a possible outcome measure. In 2024, it was used 17,248 times across 163 RCCs (30).

EUROHIS-QOL. The EUROHIS-QOL (31) consists of eight items, rated on a 5-point Likert scale, using different response options. Items include: “How would you rate your quality of life?” (response options ranging from “very poor” to “very good”); and “How satisfied are you with your personal relationships?” (response options ranging from “very dissatisfied” to “very satisfied”). The EUROHIS-QOL is one of the most widely used measures of quality of life. It was derived from the World Health Organization Quality of Life (WHOQOL) project in 2003 (32), and can be used to calculate quality-adjusted life years (QALYs). This is an important metric for evaluating the cost-effectiveness of services. Its eight items touch upon all four of SAMHSA's dimensions of recovery: health, home, purpose, and community, albeit in less specific ways (e.g., “How satisfied are you with your health?” is asked, which does not provide much insight into the degree to which persons are making informed, healthy choices that support physical and emotional wellbeing). Mental health is not specifically highlighted by the EUROHIS-QOL.

Substance Use Recovery Evaluator (SURE). The SURE (33) was developed through a rigorous, iterative process involving multiple key community groups, including both persons with SUD and SUD clinicians in the United Kingdom in 2014–2016. The SURE total score reflects substance use, the ability to cope with stressors without substance use, self-care, relationships, material resources and outlook on life. As such, this measure encompasses all four dimensions highlighted by SAMHSA: health (substance use, coping without substance use, self-care), home (material resources), purpose (outlook on life) and community (relationships). It consists of 26 items, with considerable detail on self-care and relationships. Items are rated on a 5-point Likert scale, using different response options. Examples pertaining to SAMHSA's four dimensions of recovery (i.e., health, home, purpose, and community) are as follows: Regarding substance use (i.e., pertaining to “health”), items include: “I have drunk too much” (response options ranging from “never” to “every day”). Regarding material resources (i.e., pertaining to “home”), items include: “I have had stable housing” (response options ranging from “all of the time” to “none of the time”). Regarding outlook on life (i.e., pertaining to “purpose”), items include: “I have felt positive” (response options ranging from “all of the time” to “none of the time”). Regarding relationships (i.e., pertaining to “community”), items include: “I have been treated with respect and consideration by people around me” (response options ranging from “all of the time” to “none of the time”). This outcome measure is increasingly being used by scientists and RCC directors. Among scientists, the SURE was included as an outcome measure in five recent randomized clinical trials (34–38). Among RCC directors, it has become known recently. It was added to the RDP in July 2022. In 2024, it was used 1,008 times across 163 RCCs (30).

PERMA Profiler. The PERMA Profiler (39) consists of 22 items, which assesses flourishing across five dimensions: positive emotions (experiencing joy, optimism, contentment), engagement (experiencing “flow”, feeling deeply engaged in activities), relationships (feeling valued, loved and supported), meaning (experiencing purpose in life), and accomplishments (having goals and making progress toward them). These five dimensions have direct relevance to three out of the four SAMHSA dimensions of recovery (i.e., health, purpose, and community). Other items are geared toward assessing flourishing more broadly. All items are rated on 10-point scales, using different anchors. Regarding positive emotions (i.e., pertaining to “health”), items include: “In general, how often do you feel positive?” (where 1 = “not at all” and 10 = “always”). Regarding positive emotions (i.e., in this case, pertaining to “purpose”), items include: “In general, to what extent do you lead a purposeful and meaningful life?” (where 1 = “not at all” and 10 = “completely”). Regarding relationships (i.e., pertaining to “community”), items include: “To what extent do you feel loved?” (where 1 = “not at all” and 10 = “completely”). Items assessing engagement have relevance to the recreational activities RCCs provide. Items include: “How often do you lose track of time while doing something you enjoy?” (where 1 = “never” and 10 = “always”). Also noteworthy are items that assess progress toward life goals, in line with the direct support RCCs provide: “How much of the time do you feel you are making progress toward accomplishing your goals?” (where 1 = “never” and 10 = “always”). We included this measure in this study because an important theme of the recovery movement is to think of recovery not as the absence of disease but of moving toward flourishing. The PERMA-Profiler is a widely used measure of flourishing (cited over 2,100 times in the academic literature) that comes out of the positive psychology literature, which has substantial synergy with the recovery movement (40). RCCs, which grew out of the recovery movement (41), embrace the emphasis on flourishing in a multitude of ways (e.g., fostering a caring social environment, providing opportunities for leisure activities, providing support and guidance on achieving goals, etc.), thereby making the assessment of flourishing relevant to understanding the outcomes of participating in an RCC.

Life Goals. This measure is a single question with 12 “check all that apply” response options: “In the past 3 months, has your life improved in any of these aspects?” The response options were: (1) “employment”, (2) “income”, (3) “housing”, (4) “education”, (2) “social connections”, (6) “family”, (7) “physical health”, (8) “fitness (e.g., exercise, diet)”, (9) “mental health”, (10) “spiritual well-being”, (11) “criminal justice issues”, and (12) “community involvement”. This question and its answer choices were developed by our investigative team, which included diverse perspectives: people in recovery, recovery leaders, people with experience evaluating RCCs (i.e., via ARCO certification process, via state-funding review processes), recovery scientists (psychology, health services), a prescriber of medications for opioid use disorder, and SUD clinicians. This item and its answer choices are congruent with NIDA's and SAMHSA's definition of recovery, specifically addressing this piece of the definition: “people improve their health and wellness, live self-directed lives, and strive to reach their full potential”.

### Analysis

We calculated summary statistics for the items describing the participating RCCs, and the questions we asked RCC directors about evaluating the presented outcome measures. Missing data were rare. The only questions with more than 5% missing answers were the questions where RCC directors were asked to specify the “worst” (11% of RCC directors skipped this item) and “best” measure (6%).

To explore whether RCC characteristics were related to RCC directors' evaluations of the proposed measures, we conducted exploratory univariate logistic regression analyses. In these analyses, we were constrained to examine measures that showed a minimum threshold of variation in RCC directors' evaluations. Otherwise, the statistical models cannot converge. Thus, we only conducted these analyses for measures where 10% or more of RCC directors endorsed “no” for the question: “Do you think this measure would be useful to demonstrate the impact of your RCC?” For these measures, we conducted univariate logistic regression where the dependent variable was “Do you think this measure would be useful to demonstrate the impact of your RCC?” (modeling the probability that RCC directors indicated “no”), and the univariate predictor variables were the variables describing the RCCs' physical setting, size and model of care, as described in Table 2, using *p* < 0.05. We also examined r-square values as a more descriptive evaluation of the relationship of these predictors with the dependent variables.

**Table 2:** Participating RCCs.

**Variables describing RCCs**	**Total**	
	***n*** = **114**	
	**M/%**	**(SD/** ***n*** **)**
**Physical Setting**		
Rurality (% describing primary catchment area of RCC as rural)	21.1	(24)
Has state-level funding (% yes)	58.8	(67)
**US Census region (% located in)**		
Northeast	39.5	(45)
Midwest	21.1	(24)
South	26.3	(30)
West	13.2	(15)
**RCC size and model of care**		
**RCC Staffing, M(SD)**		
Number of paid staff	8.9	(10.7)
Number of volunteer staff at your RCC:	10.2	(11.7)
**RCC Footprints** ***(in medians, due to skew)***		
Number of RCC members last year	530	
Number of active RCC members last month	125	
**RCC Model of Care** ***(% of RCCs)***		
An information-oriented place where people are connected with resources and learn more about recovery	92.1	(105)
A service-oriented place where people use services hosted by the RCC	88.6	(101)
A social place where people go to meet and spend time with peers	78.1	(89)
**Demographics of people served by the RCCs (estimated by RCC director, M(SD) across RCCs)**		
**Age** ***(% of RCC members in each age group)***		
< 18 years of age	2.3	(5.9)
18–24 years	21.3	(15.5)
25–59 years	63.1	(18.5)
60+ years	12.7	(11.1)
**Gender** ***(% of RCC members in each category)***		
Male	54.7	(14.0)
Female	42.8	(12.8)
Other	1.9	(3.5)
Ethnicity (% Hispanic)	21.0	(21.1)
**Race** ***(% of RCC members in each group)***		
American Indian or Alaskan Native	2.3	(4.1)
Asian	1.6	(2.8)
Black/African American	22.3	(20.8)
Native Hawaiian or Pacific Islander	1.1	(5.9)
White	62.7	(24.0)
More than one race	10.3	(10.9)

We counted write-in responses and summarized them by theme. Initial themes were identified by one author (BH). Three independent coders (AW, DF, and AF) then coded each write-in responses as belonging to this grouping or not (yes/no). The team iteratively added themes and re-coded write-in responses as needed by consensus.

## Results

### Description of RCCs

RCCs of which the director participated in part 2 of the survey are described in Table 2. RCCs were staffed, on average, by 8.9 paid staff members, and 10.2 volunteers. RCCs engaged a median of 530 participants per year, with a median of 125 participants engaging in activities at the RCC during the last month. The directors of the participating RCCs described their RCCs as providing informational (92%), service (89%), and social (78%) support. RCCs engaged participants of varying ages, including young adults (average of 23% of participants across RCCs) and adults aged 60+ years of age (average of 13% of participants across RCCs). Nearly half of the participants were female (average of 43% participants across RCCs), and 2% neither male nor female. RCCs engaged participants of diverse racial and/or ethnic backgrounds, including Hispanic (average of 21% of participants across RCCs) and Black participants (average of 22% of participants across RCCs).

### Evaluation of proposed outcome measures

In terms of capturing recovery progress over time (Table 3), the vast majority of RCC directors felt the presented outcome measures would be able to capture progress over time. That is, for each of the presented measures, 86% or more of RCC directors responded “yes” to the question “Do you think that participants at your RCC would show progress on this measure?”. Note that we did not ask this question for two out of the seven presented measures, because these measures cannot be assessed prior to participating in the RCC (i.e., Client Satisfaction Scale, Ways in Which RCCs Confer Benefit).

**Table 3:** Evaluation of proposed outcome measures.

**Proposed outcome measure**	**Do you think that participants at your RCC would show progress on this measure?**		**Do you think this measure would be useful to demonstrate the impact of your RCC?**	
	**% yes**	**(** ***n*** **)**	**% yes**	**(** ***n*** **)**
Ways in Which RCCs Confer Benefit	n/a		96.5	(110)
Life Goals	100.0	(114)	94.7	(108)
BARC-10	100.0	(114)	94.7	(108)
Client Satisfaction Scale (CSQ-8)	n/a		92.1	(105)
EUROHIS-QOL	92.1	(105)	89.5	(102)
Substance Use Recovery Evaluator (SURE)	93.9	(107)	83.3	(95)
PERMA profiler	86.8	(99)	72.8	(83)

In terms of being useful indicators of the impact RCCs have (Table 3), results were more mixed. For each measure, 72% or more of RCC directors answered “yes” to the question: “Do you think this measure would be useful to demonstrate the impact of your RCC?”. The measure with the most affirmative responses was our created list of “Ways in Which RCCs Confer Benefit”, which received a “yes” rating from 97% of RCC directors, followed by the Life Goals list (95% “yes”) and the BARC-10 (95% “yes”). The PERMA Profiler received the fewest “yes” ratings (73%).

### Differences in evaluations based on RCCs' physical setting, size or model of care

Our threshold of 10% or more of RCC directors endorsing “no” for the question: “Do you think this measure would be useful to demonstrate the impact of your RCC?” was met for three measures: the EUROHIS-QOL (10.5%), SURE (16.7%) and PERMA (27.2%). Logistic regression analyses (see Supplementary Tables 1–3) did not identify any significant univariate predictors of answering “no” to this question. Max r-scaled r-square values ranged from 0.00 to 0.05 for the EUROHIS-QOL and the SURE, and 0.00 to 0.03 for the PERMA. The variable that came closest to showing a statistically significant relationship to RCC directors' evaluations were “Number of active RCC members last month” (*p* = 0.09 for EUROHIS-QOL, *p* = 0.07 for SURE). All other *p* values were 0.18 or larger.

### Comments on measures

#### CSQ-8

Three RCC directors (3%) indicated that the CSQ-8 measure would not be useful to demonstrate the impact of their RCC. They offered concerns that in using this measure, “services” would need to be very carefully defined, that this measure would not capture the supportive atmosphere that is a key ingredient of the support RCCs provide, and they worried that this measure would not provide scientifically useful data.

#### Ways in which RCCs confer benefits

No RCC director (0%) answered “no” to the question: “Do you think this measure would be useful to demonstrate the impact of your RCC?”. Four RCC directors did not answer this question; for these non-responses, no write-ins were provided to comment on why this measure would not be useful. Overall, RCC directors provided 48 comments regarding additional items that could be added to this list. Many of these comments (*n* = 20, 42%) said that no further items were needed, and endorsed the list with comments like “This is a wonderful list. I can't think of others to add” and “[I] think you covered everything”. Other write-ins suggested adding items to capture additional positive impacts RCCs can have. Suggestions included adding items to capture that RCCs help develop social networks, show that there are multiple pathways to recovery, provide opportunities to “give back” and participate in advocacy and leadership, could be counted on no matter what, were culturally respectful, provided hands-on support in numerous ways, and were a safe space to ask questions and feel valued.

#### BARC-10

Five RCC directors (4%) indicated that the BARC-10 measure would not be useful to demonstrate the impact of their RCC. They offered concerns that this measure does not capture the RCC's impact on its surrounding community, is not sensitive enough to measure growth over time, and it would be difficult to attribute gains on the BARC-10 unequivocally to the RCC.

#### EUROHIS-QOL

Seven RCC directors (6%) indicated that their participants would not show progress on the EUROHIS-QOL, and nine RCC directors (8%) indicated that this measure would not be useful to demonstrate the impact of their RCC. Regarding its inability to show progress, RCC directors shared concerns that the items are not specific to recovery, they are unsure how RCC participants would react to these questions, that questions about money would be ill-placed because money is challenging early in recovery, that struggle with contentment may impact answers, that recovery is more than mental health, and that the items of the EUROHIS-QOL may not be relevant. Regarding its inability to demonstrate impact of their RCC, comments were similar. They centered on the generality of the questions, which would make it difficult to tie these ratings to participation in the RCC; thus, they felt that other measures would be better.

#### Substance Use Recovery Evaluator (SURE)

Four RCC directors (4%) indicated that their participants would not show progress on the SURE, and 17 RCC directors (15%) indicated that this measure would not be useful to demonstrate the impact of their RCC. Regarding its inability to show progress, the strongest concern was that RCC participants would not be willing to go through this measure because of its length (i.e., 26 questions). The other concern was that such a measure would be more fitting for a treatment environment as it felt diagnostic. Regarding its lack of utility to demonstrate the impact of an RCC, the strongest concern was once again its length (expressed by 7 RCC directors), followed by the impression that this measure is too diagnostic (expressed by 4 RCC directors), and focused too much on substance use (expressed by 3 RCC directors; 6 of the 16 items referred to substance use). Other comments were that the SURE would be too much for an RCC member if they had to complete it at the first visit to an RCC, that it needs more focus on coping and skills, and that the British English terminology was a barrier.

#### PERMA profiler

Ten RCC directors (9%) indicated that their participants would not show progress on the PERMA Profiler, and 26 RCC directors (23%) indicated that this measure would not be useful to demonstrate the impact of their RCC. Regarding its inability to show progress, the strongest concern centered on the length of this measure (i.e., 22 items, presented in 8 “blocks”), a concern expressed by 6 RCC directors. Five RCC directors expressed that this measure felt too clinical, does not connect to the work of RCCs, and is too complicated for RCC members. Additionally, concerns were expressed that the PERMA Profiler does not provide useful information (expressed by 4 RCC directors), the PERMA Profiler items set too high of a bar for RCC participants to achieve, the constructs measured are too broad, the measure does not focus on the basics of recovery, is not relevant to peer support, comes across as mental health focused, is too subjective and repetitive and is difficult to score.

#### Life goals

Four RCC directors (4%) indicated that the “Life Goals” measure would not be useful to demonstrate the impact of their RCC. They offered concerns that this measure is too broad, does not capture the RCC's impact on its surrounding community, is too directive (i.e., RCC staff may suggest directions but ultimately each RCC participant chooses to pursue their own goals and should not be held accountable for working toward specific goals), and is redundant with the information already reported for the Government and Performance Results Act.

### Selecting “best” and “worst” of the suggested outcome measures

In selecting the overall “best” and “worst” outcome measure among the presented measures, RCC directors were split (Table 4). Each measure was selected at least by a few RCC directors as the “best” measure, and no measure was spared the designation of the “worst” measure. Regarding the “best” measure, the most frequently selected measure was the BARC-10, which was deemed the “best” measure of the positive impact RCCs can make on people seeking or in recovery by 26% of the RCC directors, followed by our self-made list of “Ways in which RCCs Confer Benefits”, selected by 20% of RCC directors. Regarding the “worst” measure, the PERMA Profiler stood out for being selected as the “worst” measure by 25% of the RCC directors, followed by a near-tie of the CSQ-8 (14%), the Life Goals list (13%), and the SURE (13%).

**Table 4:** Selecting the “best” and “worst” outcome measure to measure RCC effectiveness.

**Proposed outcome measure**	**Which one do you think is the best measure of the positive impact RCCs can make on people seeking or in recovery?**		**Which measure do you think is the worst measure of the positive impact RCC can make on people seeking or in recovery?**	
	**% yes**	**(** ***n*** **)**	**% yes**	**(** ***n*** **)**
BARC-10	26.3	(30)	7.0	(8)
Ways in Which RCCs Confer Benefit	20.2	(23)	7.0	(8)
Client Satisfaction Scale (CSQ-8)	16.7	(19)	14.0	(16)
Life Goals	14.9	(17)	13.2	(15)
EUROHIS-QOL	7.0	(8)	9.6	(11)
Substance Use Recovery Evaluator (SURE)	6.1	(7)	13.2	(15)
PERMA Profiler	2.6	(3)	25.4	(29)

## Discussion

This study was designed to capture the perspectives of RCC directors on measures that currently exist and might feasibly be selected as an outcome measure for research studies seeking to test the effectiveness of RCCs. This issue is of critical importance to move the field forward. The promise and importance of recovery support services is highlighted in the National Drug Control Strategy, which in 2022 included recovery support services for the first time within its plan to address the overdose crisis and to support recovery (5). This emphasis on recovery support services remains intact in the 2025 Statement of Drug Policy Priorities (42). Yet to date, research on recovery support services disproportionally consists of smaller scale studies and site-specific reports (43). One of the recovery support services in particular need of more research are RCCs (44). To date, no RCT has been conducted to test the effectiveness of RCCs, or, indeed, any portion of it. Identifying a suitable outcome measure for such an RCT is necessary in order to conduct an RCT that will provide valid insight into the effectiveness of RCCs.

Overall, our results indicate that there is substantial buy-in from RCC directors for existing scales that could be used to capture positive impacts of an RCC on its participants. All of the suggested outcome measures were deemed useful by the majority of RCC directors. Even the least endorsed measure, the PERMA Profiler, was deemed useful by more than 70% of the RCC directors. Several scales, which ranged quite widely in their conceptualization of “effectiveness” of RCCs (i.e., in terms of satisfaction, achievement of life goals, concrete ways in which RCCs helped, and attainment of recovery capital), reached very high levels of consensus with more than 90% of RCC directors deeming them useful. Furthermore, our exploratory analyses suggest that RCC directors' evaluations were not related to RCCs physical setting, size or model of care. This level of agreement is encouraging, as it suggests that there may be multiple tools that researchers can use to capture the effectiveness of RCCs on individuals that ring true to RCC directors. Results also suggest that these outcome measures are in sync with how RCCs are designed and meant to confer benefit.

At the same time, RCC directors shared important concerns that can further guide the selection and design of valid outcome measures for effectiveness research on RCCs. RCC directors expressed the importance of keeping self-report measures short and relevant. They emphasized that measures should not be overly focused on mental health and should certainly not have a diagnostic feel to them. These notes RCC directors provided for the specific scales can help researchers refine these tools, and understand their limitations.

Surprisingly well-liked was the scale our team put together, the “Ways in which RCCs Confer Benefits”. This scale does not have a long history of psychometric evaluation, which many of the other scales did. Rather, for this scale, the current study represented a very early step in the scale development process: item generation (45). Our team, spanning people with diverse touchpoints with RCCs, generated the first set of items, and with the input from RCC directors across the US, we now have a more complete set. The next step would be to evaluate the intercorrelation of items to reduce redundancy and understand potentially existing sub-factors. Findings from this study suggest that such work would be a worthwhile endeavor. This scale resonated strongly with RCC directors. That said, this scale is not a measure of recovery: it does not measure the degree to which “people improve their health and wellness, live self-directed lives, and strive to reach their full potential”, which is SAMHSA's and NIDA's definition of recovery. But perhaps this concrete list of “Ways in which RCCs Confer Benefits” could be used as a process measure, which would capture the immediate impacts of participating in an RCC. This short-term outcome would be in line with NIDA's perspective that “recovery support services are thought to assist individuals with the recovery process” (as stated in RFA-DA-20-014), as this measure would assess very concretely how RCCs assist individuals in their recovery process. Perhaps this measure could also be useful to RCCs in their routine data collection and program evaluation procedures as a way to report immediate and concrete impacts of their work. Such reporting would go beyond a more standard process evaluation that reports on the number and types of encounters and services (46), and might potentially be a useful datapoint to include when pursuing funding opportunities to sustain the operations of an RCC. Regarding research on the effectiveness of RCCs, it would be important to evaluate to what degree these short-term achievements relate to downstream, longer-term outcomes, such as life goals (also highly endorsed by RCC directors) and recovery outcomes, as measured by the SURE.

The second major finding of our study was that there was no clear consensus on which proposed outcome measure was the best. This finding at first felt disappointing, as it would have been very nice indeed to be able to conclude that one outcome measure was by far more suitable than the others. The urgency for identifying such a singular outcome is high, because the standard design of an RCT demands the choosing of a single primary outcome measure. It is performance on this singular outcome measure that typically defines the “success” or “failure” of an RCT. Reality, however, is more complex. Indeed, the addiction field has a long history of debating about appropriate outcomes with achieving minimal consensus (47). To us, this finding suggests that in designing a trial to assess the effectiveness of RCCs, the selection of the outcome measure needs to done in partnership and careful deliberation with the RCCs participating in that study. Each RCC is unique, and while there is a strong, robust overall service model of RCCs (2), RCCs may interpret effectiveness in different ways based on their unique goals, client demographics, geography, or capacity. Thus, the selected outcome measure needs to reflect the specific areas of strengths of the RCCs whose effectiveness is being measured. It is our hope that the scales we examined in this study may provide a solid starting place, as all of the measures we presented to RCC directors had considerable buy-in from RCC directors.

### Limitations

When interpreting the findings of our study, some limitations should be kept in mind. First, there are limits to the generalizability of these findings. In this study, we attempted to reach the RCC directors of all RCCs nationwide. We succeeded in obtaining feedback on the suggested measure from 58% of RCC directors of the 198 RCCs identified nationwide. Because we have no systematic data on the RCCs that declined to participate in our survey, we do not know if specific factors were related to RCCs choosing to participate in our study. Moreover, we also do not know that the 198 identified RCCs nationwide were indeed the total number of RCCs in existence at that time. While our outreach methods were multi-layered and robust, we may have missed some RCCs. Moreover, more RCCs may have opened their doors since we conducted this study, and their voices are not represented in these data.

Second, we only selected a handful of plausible outcome measures when many more could have been examined. Our small selection of scales was driven by the need to keep the survey doable. We were worried that survey fatigue would impede the review and evaluation of more scales. One of the dimensions we did not assess with our suggested measures was the degree to which RCCs provide belonging and access to cultural community resources, which is an important component of recovery for some (48). We hope that future research efforts will consider this component when selecting outcome measures for effectiveness studies of RCCs.

Third, the measures we presented focused on the efficacy of RCCs to support the recovery journey of their members. We did not ask about “reach”, which is a critically important aspect of the effectiveness of services (i.e., effectiveness = efficacy ^*^ reach) (49). Regarding reach, prior research has suggested that RCCs are particularly promising in reaching young adults and BIPOC communities (2). In understanding the effectiveness of RCCs, however, it will be important to measure concretely the degree to which they are able to reach the populations they seek to reach, including their ability to overcome language barriers for Hispanic/Latino populations, for example.

Fourth, in asking RCC directors to evaluate the measures' sensitivity to assess change (i.e., “Do you think that participants at your RCC would show progress on this measure”), we relied quite substantially on their intuition. This is a very challenging question to answer, and certainly, the best way to answer it is empirically by testing if RCC participants demonstrate changes over time on these measures. Note also that RCC directors and RCC participants may have different views on the relevance of the measures we evaluated in this study. In studies evaluating clinical services, it has been shown that patients have distinctive perspectives when compared to providers regarding definitions and ratings of quality (50). Regarding opioid use disorder in particular, patients and clinicians have expressed some divergent views regarding the “success” of treatment (51). Notably, they agreed on many points (i.e., that OUD treatment “success” needs to include in its conceptualization an appraisal of relationships, wellbeing, role-functioning), while differing on fewer points (e.g., conceptualizing tapering off medications and feeling decreased shame as a marker of success) (51). We believe that discrepancies between RCC directors and RCC participants in evaluating the relevance of RCC outcome measures to be less pronounced, because RCC directors have lived experience of recovery, and often started by being RCC members themselves. Moreover, in asking RCC directors to evaluate the proposed outcome measures, our goal was to identify measures that would not pass an initial test of reflection, so as to rule-out measures that based on RCC directors' experience would not be a good outcome measure to pursue. Future studies that assess these measures over time in people who participate in RCCs will show to what degree these expectations and intuitions bear out.

Fifth, regarding the ability of our study to rule out “bad” measures, it is worth noting that it may have been easy for RCC directors to endorse these measures, as they did not need to consider participant fatigue, how and when to implement these measures, or how to develop systems to meaningfully utilize such data. They knew that our question was a hypothetical question, designed to find out what measures scientists should consider using in studies to assess the effectiveness of RCCs.

Sixth, data were collected in 2021–2022, which seems a short time ago, but this is a time of transformative change and rapid expansion of peer recovery support services in general and RCCs in particular. New model standards are emerging for the peer recovery workforce (52), and greater collaboration between clinical settings and RCCs are recommended (53). These shifts may well impact how RCCs operate. New measures may emerge that are better suited to capture the impact of RCCs. Our findings provide a starting point, which we are offering with the hope of keeping the voices and thoughts of RCC directors front and center in the discussion of how to define and determine the effectiveness of RCCs.

### Future Directions

The limitations of our study highlight the important next steps that need to be taken to move the research on the effectiveness of RCCs forward. To summarize, research is needed that examines the sensitivity to change of these suggested measures, as measured by asking RCC participants to complete these measures over time (i.e., in longitudinal observational studies or clinical trials). Research is also needed to test if theorized causal links exist between changes in process measures to changes in downstream outcomes. Additionally, although our initial exploratory analyses did not reveal difference by RCCs' physical setting, size or model of care, this is still an area that would be important to investigate further. Moreover, new ideas and research need to be generated to be able to study the impact of RCCs not just on the individual level but also on the community level.

## Conclusion

Our findings indicate that several existing scales resonate with RCC directors, and thus may be useful in research seeking to determine the effectiveness of RCCs, as either process or outcome variables. Identifying a single primary outcome measure for a large scale RCT remains challenging, however, as there was little consensus on what scale would be the best scale to measure the positive impacts of RCCs. These findings point to the importance of developing large-scale RCTs to test the effectiveness of RCC in partnership with RCCs, so as to ensure that the design of the study, especially the selection of its primary outcome measure, is congruent with how RCCs function and seek to confer benefit. Such close collaboration is especially needed given the current rapidly changing landscape in which RCCs operate.

## Data Availability

The raw data supporting the conclusions of this article will be made available by the authors, without undue reservation.

## References

[1] White WL Kelly JF Roth JD. New addiction-recovery support institutions: mobilizing support beyond professional addiction treatment and recovery mutual aid. J Groups Addict Recover. (2012) 7:297–317. 10.1080/1556035X.2012.705719

[2] Hoeppner BB Simpson HV Weerts C Riggs MJ Williamson AC Finley-Abboud D. A nationwide survey study of recovery community centers supporting people in recovery from substance use disorder. J Addict Med. (2024) 18:274–81. 10.1097/ADM.0000000000001285 38426533 PMC11150096

[3] Valentine P. “Peer-based recovery support services within a recovery community organization: the CCAR experience”. In: Kelly JF White WL Kelly JF White WL, editors. *Addiction Recovery Management: Theory, Research and Practice*. Humana Press (2011). p. 259–79. Current clinical psychiatry. 10.1007/978-1-60327-960-4_14

[4] Kelly JF Bergman B Hoeppner BB Vilsaint C White WL. Prevalence and pathways of recovery from drug and alcohol problems in the United States population: implications for practice, research, and policy. Drug Alcohol Depend. (2017) 181:1629. 10.1016/j.drugalcdep.2017.09.028 29055821 PMC6076174

[5] United States. National Drug Control Strategy. Washington, DC: Office of National Drug Control Policy (2022).

[6] National Institute of Health. Recovery Research Networks (2025).

[7] Gormley MA Pericot-Valverde I Diaz L Coleman A Lancaster J Ortiz E. Effectiveness of peer recovery support services on stages of the opioid use disorder treatment cascade: a systematic review. Drug Alcohol Depend. (2021) 229:109123. 10.1016/j.drugalcdep.2021.109123 34700201

[8] Holtrop JS Estabrooks PA Gaglio B Harden SM Kessler RS King DK. Understanding and applying the RE-AIM framework: clarifications and resources. J Clin Trans Sci. (2021) 5:e126. 10.1017/cts.2021.789 34367671 PMC8327549

[9] Judd H Meier CL Yaugher AC Campbell E Atismé-Bevins K. Opioid use disorder stigma reduction through story telling narrative and sharing: a qualitative review and lessons learned. Int J Ment Health Addict. (2023) 21:468–83. 10.1007/s11469-021-00606-y

[10] Biondi BE Zheng X Frank CA Petrakis I Springer SA. A literature review examining primary outcomes of medication treatment studies for opioid use disorder: What outcome should be used to measure opioid treatment success? Am J Addict. (2020) 29:249–67. 10.1111/ajad.13051 32346932 PMC7377168

[11] Amato L Minozzi S Davoli M Vecchi S. Psychosocial combined with agonist maintenance treatments versus agonist maintenance treatments alone for treatment of opioid dependence. Cochrane Database Syst Rev. (2011) CD004147. 10.1002/14651858.CD004147.pub4 21975742

[12] Day E Mitcheson L. Psychosocial interventions in opiate substitution treatment services: does the evidence provide a case for optimism or nihilism? Addiction. (2017) 112:1329–36. 10.1111/add.13644 28044376

[13] Kelly JF Hoeppner B. A biaxial formulation of the recovery construct. Addict Res Theory. (2015) 23:5–9. 10.3109/16066359.2014.930132

[14] Fan AZ Chou SP Zhang H Jung J Grant BF. Prevalence and correlates of past-year recovery from DSM-5 alcohol use disorder: results from National Epidemiologic Survey on Alcohol and Related Conditions-III. Alcohol Clin Exp Res. (2019) 43:2406–20. 10.1111/acer.14192 31580502

[15] Witkiewitz K Wilson AD Pearson MR Montes KS Kirouac M Roos CR. Profiles of recovery from alcohol use disorder at three years following treatment: can the definition of recovery be extended to include high functioning heavy drinkers? Addiction. (2019) 114:69–80. 10.1111/add.14403 30063267 PMC6289769

[16] Witkiewitz K Tucker JA. Abstinence not required: expanding the definition of recovery from alcohol use disorder. Alcohol Clin Exp Res. (2020) 44:36. 10.1111/acer.14235 31709568 PMC6980896

[17] Rosenberg H Grant J Davis AK. Acceptance of non-abstinence as an outcome goal for individuals diagnosed with substance use disorders: a narrative review of published research. J Stud Alcohol Drugs. (2020) 81:405–15. 10.15288/jsad.2020.81.405 32800076

[18] Davidson L White W. The concept of recovery as an organizing principle for integrating mental health and addiction services. J Behav Health Serv Res. (2007) 34:109–20. 10.1007/s11414-007-9053-7 17351758

[19] Substance Abuse Mental Health Services Administration. SAMHSA's Working Definition of Recovery. Author Rockville, MD (2012).

[20] Donovan DM Bigelow GE Brigham GS Carroll KM Cohen AJ Gardin JG. Primary outcome indices in illicit drug dependence treatment research: Systematic approach to selection and measurement of drug use end-points in clinical trials. Addiction. (2012) 107:694–708. 10.1111/j.1360-0443.2011.03473.x 21781202 PMC3537825

[21] Tiffany ST Friedman L Greenfield SF Hasin DS Jackson R. Beyond drug use: a systematic consideration of other outcomes in evaluations of treatments for substance use disorders. Addiction. (2012) 107:709–18. 10.1111/j.1360-0443.2011.03581.x 21981638 PMC3257402

[22] Del Boca FK Darkes J. ‘Nothing is more practical than a good theory': outcome measures in addictions treatment research. Addiction. (2012) 107:719–20. 10.1111/j.1360-0443.2011.03647.x 22372698

[23] Moore GF Audrey S Barker M Bond L Bonell C Hardeman W. Process evaluation of complex interventions: Medical Research Council guidance. BMJ. (2015) 350:h1258. 10.1136/bmj.h1258 25791983 PMC4366184

[24] Harris PA Taylor R Thielke R Payne J Gonzalez N Conde JG. Research electronic data capture (REDCap)—a metadata-driven methodology and workflow process for providing translational research informatics support. J Biomed Inform. (2009) 42:377–81. 10.1016/j.jbi.2008.08.010 18929686 PMC2700030

[25] Larsen DL Attkisson CC Hargreaves WA Nguyen TD. Assessment of client/patient satisfaction: development of a general scale. Eval Program Plann. (1979) 2:197–207. 10.1016/0149-7189(79)90094-6 10245370

[26] Vilsaint CL Kelly JF Bergman BG Groshkova T Best D White W. Development and validation of a Brief Assessment of Recovery Capital (BARC-10) for alcohol and drug use disorder. Drug Alcohol Depend. (2017) 177:71–6. 10.1016/j.drugalcdep.2017.03.022 28578224

[27] Groshkova T Best D White W. The Assessment of Recovery Capital: properties and psychometrics of a measure of addiction recovery strengths. Drug Alcohol Rev. (2013) 32:187–94. 10.1111/j.1465-3362.2012.00489.x 22882622

[28] Bunaciu A Bliuc A-M Best D Hennessy EA Belanger MJ Benwell CS. Measuring recovery capital for people recovering from alcohol and drug addiction: a systematic review. Addict Res Theory. (2024) 32:225–36. 10.1080/16066359.2023.2245323 39045096 PMC11262562

[29] Granfield R Cloud W. Coming Clean: Overcoming Addiction Without Treatment. New York, NY: NYU Press (1999).

[30] Riggs MJ Picard J. Exploratory Analysis of the Recovery Data Platform to Uncover Group Associations Between Engagement & Outcomes in RCC Participants. presented at: Monthly online seminar series of the NIDA-funded infrastructure-building initative on Advancing the Science on Recovery Community Centers (RCCs) (2025). https://www.healththroughflourishing.com/seminar-series. Session 42. (Accessed July 7, 2025).

[31] Schmidt S Mühlan H Power M. The EUROHIS-QOL 8-item index: psychometric results of a cross-cultural field study. Eur J Public Health. (2006) 16:420–8. 10.1093/eurpub/cki155 16141303

[32] Group TW. The World Health Organization quality of life assessment (WHOQOL): development and general psychometric properties. Soc Sci Med. (1998) 46:1569–85. 10.1016/S0277-9536(98)00009-4 9672396

[33] Neale J Vitoratou S Finch E Lennon P Mitcheson L Panebianco D. Development and validation of ‘SURE': a patient reported outcome measure (PROM) for recovery from drug and alcohol dependence. Drug Alcohol Depend. (2016) 165:159–67. 10.1016/j.drugalcdep.2016.06.006 27344196 PMC4946826

[34] Kelly PJ Deane FP Baker AL Townsend C McKay JR Degan TJ. The continuing care project: a multi-arm randomised controlled trial of a continuing care telephone intervention following residential substance use treatment. Drug Alcohol Depend. (2025) 272:112668. 10.1016/j.drugalcdep.2025.112668 40288058

[35] Marsden J Kelleher M Gilvarry E Mitcheson L Bisla J Cape A. Superiority and cost-effectiveness of monthly extended-release buprenorphine versus daily standard of care medication: a pragmatic, parallel-group, open-label, multicentre, randomised, controlled, phase 3 trial. EClinicalMedicine. (2023) 66:102311. 10.1016/j.eclinm.2023.102311 38045803 PMC10692661

[36] Lintzeris N Dunlop AJ Haber PS Lubman DI Graham R Hutchinson S. Patient-reported outcomes of treatment of opioid dependence with weekly and monthly subcutaneous depot vs daily sublingual buprenorphine: a randomized clinical trial. JAMA Network Open. (2021) 4:e219041. 10.1001/jamanetworkopen.2021.9041 33970256 PMC8111483

[37] Vanderijst L Hever F Buot A Dauré C Benoit J Hanak C. Psilocybin-assisted therapy for severe alcohol use disorder: protocol for a double-blind, randomized, placebo-controlled, 7-month parallel-group phase II superiority trial. BMC Psychiatry. (2024) 24:77. 10.1186/s12888-024-05502-y 38279085 PMC10821548

[38] Saur S Weisel KK Lang C Fuhrmann LM Steins-Loeber S Enewoldsen N. App-based maintenance treatment for alcohol use disorder after acute inpatient treatment: Study protocol for a multicentre randomized controlled trial. Internet Interv. (2022) 28:100517. 10.1016/j.invent.2022.100517 35251940 PMC8888955

[39] Butler J Kern ML. The PERMA-profiler: a brief multidimensional measure of flourishing. Int J Wellbeing. (2016) 6, 1–48. 10.5502/ijw.v6i3.52638015927

[40] Krentzman AR. Review of the application of positive psychology to substance use, addiction, and recovery research. Psychol Addict Behav. (2013) 27:151. 10.1037/a0029897 22985057 PMC3531570

[41] White WL. The new recovery advocacy movement in America. Addiction. (2007) 102:696–703. 10.1111/j.1360-0443.2007.01808.x 17493104

[42] The White House. Statement of Drug Policy Priorities (2025).

[43] Jason LA Salomon-Amend M Guerrero M Bobak T O'Brien J Soto-Nevarez A. The emergence, role, and impact of recovery support services. Alcohol Res. (2021) 41:04. 10.35946/arcr.v41.1.04 33796431 PMC7996242

[44] Gaumond P Martin LA Hagman BT Davis MJ. Substance use disorder recovery research opportunities: perspectives from a federal interagency workgroup. Front Public Health. (2025) 13:1585533. 10.3389/fpubh.2025.1585533 40276345 PMC12018379

[45] DeVellis RF Thorpe CT. Scale Development: Theory and Applications. Sage Publications (2021).

[46] Sorensen JL Llamas JD. Process evaluation of a community outpatient program treating substance use disorders. J Community Psychol. (2018) 46:844–55. 10.1002/jcop.21976 30197457 PMC6124892

[47] Donovan DM. More mice or a better mouse trap? Reflections on primary outcome indices in illicit drug dependence treatment research. Addiction. (2012) 107:723–4. 10.1111/j.1360-0443.2012.03784.x 22372701

[48] Shahid S Best D. Exploring cultural dynamics of Black Asian Minority Ethnic (BAME) women in addiction recovery: a comparison of three women from different ethnic backgrounds. Drugs Educ Prev Policy. (2024) 1–12. 10.1080/09687637.2024.2349077

[49] Glasgow RE Lichtenstein E Marcus AC. Why don't we see more translation of health promotion research to practice? Rethinking the efficacy-to-effectiveness transition. Am J Public Health. (2003) 93:1261–7. 10.2105/AJPH.93.8.1261 12893608 PMC1447950

[50] Sofaer S Firminger K Patient Patient perceptions of the quality of health services. Annu Rev Public Health. (2005) 26:513–59. 10.1146/annurev.publhealth.25.050503.153958 15760300

[51] Hooker SA Sherman MD Lonergan-Cullum M Nissly T Levy R. What is success in treatment for opioid use disorder? Perspectives of physicians and patients in primary care settings. J Subst Abuse Treat. (2022) 141:108804. 10.1016/j.jsat.2022.108804 35643586

[52] Substance Abuse and Mental Health Services Administration. SAMHSA's National Model Standards for Peer Support Certification (2023).16809591

[53] Substance Abuse and Mental Health Services Administration. TIP 64: Incorporating Peer Support Into Substance Use Disorder Treatment Services (2023). 37856637

