# Supplementary material for: How to measure the effectiveness of recovery community centers: insights gained from a nationwide survey of directors of RCCs
Source: Front Public Health. 2025 Jul 23;13:1532812. doi: 10.3389/fpubh.2025.1532812 (PMC12326742; doi:10.3389/fpubh.2025.1532812)
Supplement: Supplementary file 1 [file Table_1.pdf]

Supplementary Table 1. Univariate logistic regression results of modeling the likelihood of RCC directors finding the EUROHIS-QOL 'not useful'

	EUROHIS-QOL			
	OR	95% CI	*p*	r2
**Physical Setting**				
Rurality	0.77	[0.15, 3.82]	0.75	0.00
US Census region				0.02
Northeast	ref			
Midwest	2.05	[0.38, 11.08]	0.40	
South	1.52	[0.29, 8.09]	0.62	
West	2.10	[0.32, 13.99]	0.44	
Has state-level funding	1.25	[0.34, 4.53]	0.74	0.00
**RCC size and model of care**				
RCC Staffing, M(SD)				
Number of paid staff	0.93	[0.83, 1.05]	0.27	0.04
Number of volunteer staff at your RCC:	0.98	[0.92, 1.05]	0.62	0.01
RCC Footprints *(in medians, due to skew)*				
Number of RCC members last year	1.00	[1.00, 1.00]	0.52	0.01
Number of active RCC members last month	1.00	[1.00, 1.00]	0.09	0.05
RCC Model of Care				
An information-oriented place	0.34	[0.06, 1.86]	0.21	0.03
A service-oriented place	1.35	[0.16, 11.48]	0.78	0.00
A social place	did not converge due to zero cell count			

Note: r2 = max rescaled r-square

Supplementary Table 2. Univariate logistic regression results of modeling the likelihood of RCC directors finding the SURE 'not useful'

	SURE			
	OR	95% CI	*p*	r2
**Physical Setting**				
Rurality	0.45	[0.1, 2.13]	0.31	0.02
US Census region				0.01
Northeast	ref			
Midwest	0.68	[0.16, 2.84]	0.59	
South	0.69	[0.19, 2.55]	0.58	
West	0.69	[0.13, 3.69]	0.67	
Has state-level funding	0.78	[0.28, 2.21]	0.64	0.00
**RCC size and model of care**				
RCC Staffing, M(SD)				
Number of paid staff	1.02	[0.98, 1.06]	0.35	0.01
Number of volunteer staff at your RCC:	1.01	[0.97, 1.05]	0.63	0.00
RCC Footprints *(in medians, due to skew)*				
Number of RCC members last year	1.00	[1.00, 1.00]	0.72	0.00
Number of active RCC members last month	1.00	[1.00, 1.00]	0.07	0.05
RCC Model of Care				
An information-oriented place	1.47	[0.17, 12.57]	0.72	0.00
A service-oriented place	2.31	[0.28, 19.06]	0.44	0.01
A social place	2.26	[0.48, 10.65]	0.30	0.02

Note: r2 = max rescaled r-square

Supplementary Table 3. Univariate logistic regression results of modeling the likelihood of RCC directors finding the PERMA 'not useful'

	PERMA			
	OR	95% CI	*p*	r2
**Physical Setting**				
Rurality	0.72	[0.24, 2.16]	0.55	0.01
US Census region				0.03
Northeast	ref			
Midwest	0.60	[0.18, 1.93]	0.39	
South	0.61	[0.2, 1.85]	0.39	
West	0.33	[0.07, 1.66]	0.18	
Has state-level funding	0.98	[0.4, 2.38]	0.96	0.00
**RCC size and model of care**				
RCC Staffing, M(SD)				
Number of paid staff	1.01	[0.97, 1.05]	0.60	0.00
Number of volunteer staff at your RCC:	1.00	[0.96, 1.04]	0.96	0.00
RCC Footprints *(in medians, due to skew)*				
Number of RCC members last year	1.00	[1.00, 1.00]	0.59	0.00
Number of active RCC members last month	1.00	[1.00, 1.00]	0.42	0.01
RCC Model of Care				
An information-oriented place	1.17	[0.23, 5.99]	0.85	0.00
A service-oriented place	1.74	[0.36, 8.47]	0.50	0.01
A social place	2.25	[0.61, 8.33]	0.22	0.02

Note: r2 = max rescaled r-square
