# Supplementary material for: How to measure the effectiveness of recovery community centers: insights gained from a nationwide survey of directors of RCCs
Source: Front Public Health. 2025 Jul 23;13:1532812. doi: 10.3389/fpubh.2025.1532812 (PMC12326742; doi:10.3389/fpubh.2025.1532812)
Supplement: Supplementary file 2 [file Data_Sheet_1.pdf]

Ways in Which RCCs Confer Benefit

This is not a validated scale.

Recovery community centers provide support in a number of ways. Each person is different, and each person will experience an RCC and the community it provides in a different way. One way to capture to what degree a person has felt supported through their participation in an RCC is to ask them about HOW they have felt supported.

Below is a list of items we made up that we think might capture the many different ways in which RCCs may support people in recovery during their recovery journey. To use such a descriptive list, you would ask participants at your RCC to rate the following items:

Instructions: The next questions are about your experience with your RCC. As you are evaluating these items, keep in mind all the services and elements of an RCC, including staff, resources, other participants, etc. On a scale from 1 ***(Strongly disagree)*** to 6 ***(Strongly agree)***, please indicate your level of agreement with the following statements:

The RCC I attend….

1. ... gave me confidence to persist in my recovery.
2. ... was a safe haven in a moment of crisis.
3. ... gave me trusted advice whenever I needed it.
4. ... has shown me that recovery is something I can achieve.
5. ... has shown me that recovery is worth striving for.
6. ... helped me feel supported in my recovery.
7. ... gave me a safe space to work through challenges I encounter in my recovery.
8. ... lets me feel part of a community that accepts me the way I am.
9. ... has given me access to concrete resources I need to succeed in my recovery.
10. ... has helped me navigate systems and services that can help me succeed in my recovery.
11. ... helped me become more at peace with myself.
12. ... has energized me to move forward with my life, in a way that feels good.
13. ... has provided me opportunities to give back to the community.
14. ... has given me a vision of how to navigate my recovery.
15. ... has taught me concrete steps I can take to progress with my recovery.
16. ... has added joy and friendship to my life.
17. ... keeps encouraging me to stay on track with my recovery.
