# Supplementary material for: How to measure the effectiveness of recovery community centers: insights gained from a nationwide survey of directors of RCCs
Source: Front Public Health. 2025 Jul 23;13:1532812. doi: 10.3389/fpubh.2025.1532812 (PMC12326742; doi:10.3389/fpubh.2025.1532812)
Supplement: Supplementary file 3 [file Data_Sheet_2.pdf]

Suggested Outcome Measure #1 of 7: Life Goals

One way to document the impact of an RCC is to show how many RCC participants achieve concrete goals in their life.

In order to assess this, you would ask your RCC participants the following question:

In the past three months, has your life improved in any of these aspects? Select ALL that apply.

- ☐ Employment
- ☐ Income
- ☐ Housing
- ☐ Education
- ☐ Social connections
- ☐ Family
- ☐ Physical health
- ☐ Fitness (e.g., exercise, diet)
- ☐ Mental health
- ☐ Spiritual well-being
- ☐ Criminal justice issues
- ☐ Community involvement

You would NOT ask this question when RCC participants first come to your center, but rather 3 months after their first visit, and then at systematic assessments thereafter (e.g., every 6 months).

Evaluation

Do you think that participants at your RCC would show progress on this measure (i.e., that they would check off any of these boxes)?

- ○ Yes
- ○ No

Please explain: ______________________________

Do you think this measure would be useful to demonstrate the impact of your RCC?

- ○ Yes
- ○ No

Please explain: ______________________________

Suggested Outcome Measure #2 of 7: Brief Assessment of Recovery Capital (BARC-10)

This measure, the BARC-10, is possibly the best known measure of recovery capital. It is the short version of a measure called the Assessment of Recovery Capital (ARC). The ARC (Groshkova et al, 2013) is a self-report, strength-based measure of an individual's personal and social resources that can support recovery from a substance use disorder.

To complete this measure, RCC participants would be asked to indicate the level of agreement with the following statements:

	1 - Strongly Disagree	2	3	4	5	6 - Strongly Agree
There are more important things to me in life than using substances	○	○	○	○	○	○
In general, I am happy with my life.	○	○	○	○	○	○
I have enough energy to complete the tasks I set myself.	○	○	○	○	○	○
I am proud of the community I live in and feel part of it.	○	○	○	○	○	○
I get lots of support from friends.	○	○	○	○	○	○
I regard my life as challenging and fulfilling without the need for using drugs or alcohol.	○	○	○	○	○	○
My living space has helped to drive my recovery journey.	○	○	○	○	○	○
I take full responsibility for my actions.	○	○	○	○	○	○
I am happy dealing with a range of professional people.	○	○	○	○	○	○
I am making good progress on my recovery journey.	○	○	○	○	○	○

Scoring

After participants answer these item, the scale is scored by adding together the response values for each of the ten questions. Total scores can range from 10 to 60, where 10 indicates the lowest amount of recovery capital and 60 indicates the highest amount of recovery capital.

In order to use this scale to evaluate the impact of your RCC, you would assess this scale when participants first come to you RCC, and then ask them again at a pre-determined follow-up point (e.g., 3-month later) to see if their scores have improved.

Evaluation

Do you think that participants at your RCC would show progress on this measure (i.e., their score would improve over time)?	◯ Yes<br>◯ No
Please explain:	__________________________________
Do you think this measure would be useful to demonstrate the impact of your RCC?	◯ Yes<br>◯ No
Please explain:	__________________________________

Suggested Outcome Measure #3 of 7: EUROHIS-QOL

The EUROHIS-QOL (Schmidt et al., 2006) is not specific to recovery, but rather is a widely used eight-item measure of quality of life, adapted from a measure the World Health Organization has created to measure quality of life.

In order to use this scale, you would ask participants of your RCC to rate the following items, based on their experience during the last four weeks.

	Very poor	Poor	Neither poor nor good	Good	Very good
How would you rate your quality of life?	○	○	○	○	○

	Very dissatisfied	Dissatisfied	Neither satisfied nor dissatisfied	Satisfied	Very satisfied
How satisfied are you with your health?	○	○	○	○	○

	Not at all	A little	Moderately	Mostly	Completely
Do you have enough energy for everyday life?	○	○	○	○	○

	Very dissatisfied	Dissatisfied	Neither satisfied nor dissatisfied	Satisfied	Very satisfied
How satisfied are you with your ability to perform your daily living activities?	○	○	○	○	○
How satisfied are you with yourself?	○	○	○	○	○
How satisfied are you with your personal relationships?	○	○	○	○	○
Have you enough money to meet your needs?	○	○	○	○	○
How satisfied are you with the conditions of your living place?	○	○	○	○	○

Scoring

The scale score is calculated by taking the average of responses to all 8 questions. The final score can range from 1 to 5, where 1 indicates the lowest quality of life and 5 indicates the highest.

Evaluation

Do you think that participants at your RCC would show progress on this measure (i.e., their score would improve over time)?	◯ Yes<br>◯ No
Please explain:	________________________
Do you think this measure would be useful to demonstrate the impact of your RCC?	◯ Yes<br>◯ No
Please explain:	________________________

You missed a question. If you meant to leave a question blank, please press submit to continue.

Suggested Outcome Measure #4 of 7: Substance Use Recovery Evaluator (SURE)

This scale is a newly developed scale that is a patient-reported outcome measure for assessing recovery from drug and alcohol dependency. It was developed with people in recovery in the United Kingdom. Dr. Joanne Neale recently presented on this scale as part of our seminar series:

https://www.recoveryanswers.org/addiction-research-summaries/seminar-series/

In order to use this scale to assess the impact of your RCC, you would ask participants of your RCC to complete the following questions when they first come to your RCC, and at a later timepoint.

Section A

Thinking about the last week, please rate yourself in each of the following statements.

Drinking and Drug Use (Part 1)

	Never	On 1 or 2 days	On 3 or 4 days	On 5 or 6 days	Every day
I have drunk too much	◯	◯	◯	◯	◯
I have used street drugs	◯	◯	◯	◯	◯
I have experienced cravings	◯	◯	◯	◯	◯

Drinking and Drug Use (Part 2)

	All of the time	Most of the time	A fair amount of the time	A little of the time	None of the time
I have coped with problems without misusing drugs or alcohol	◯	◯	◯	◯	◯
I have managed pains and ill-health without misusing drugs or alcohol	◯	◯	◯	◯	◯
I have been spending my free time on hobbies and interests that do not involve drugs or alcohol	◯	◯	◯	◯	◯

Section B

Still thinking about the last week, please rate yourself in each of the following statements.

Self-Care

	All of the time	Most of the time	A fair amount of the time	A little of the time	None of the time
I have been taking care of my mental health	◯	◯	◯	◯	◯

I have been taking care of my physical health	○	○	○	○	○
I have been eating a good diet	○	○	○	○	○
I have slept well	○	○	○	○	○
I have had a good daily routine	○	○	○	○	○

Relationships

	All of the time	Most of the time	A fair amount of the time	A little of the time	None of the time
I have been getting on well with people	○	○	○	○	○
I have felt supported by people around me	○	○	○	○	○
I have been treated with respect and consideration by people around me	○	○	○	○	○
I have treated others with respect and consideration	○	○	○	○	○

Material Resources

	All of the time	Most of the time	A fair amount of the time	A little of the time	None of the time
I have had stable housing	○	○	○	○	○
I have had a regular income (from benefits, work, or other legal sources)	○	○	○	○	○
I have been managing my money well	○	○	○	○	○

Outlook on Life

	All of the time	Most of the time	A fair amount of the time	A little of the time	None of the time
I have felt happy with my overall quality of life	○	○	○	○	○
I have felt positive	○	○	○	○	○
I have had realistic hopes and goals for myself	○	○	○	○	○

Section C

Still thinking about the last week, please record how important each of the following have been to you.

	Not important	A little important	Important	Very important
Reducing or abstaining from drinking or drug taking	◯	◯	◯	◯
Looking after yourself (physically taking care of yourself, mentally taking care of yourself, having a good diet, sleeping well, having a good routine)	◯	◯	◯	◯
Having good relationships with other people (getting on with people, feeling supported by people, being treated with respect, treating others with respect)	◯	◯	◯	◯
Having resources and belongings (stable housing, regular income, managing money)	◯	◯	◯	◯
Outlook on life (having a good quality of life, feeling positive, having realistic hopes and goals)	◯	◯	◯	◯

Scoring

The SURE score is calculated by sum-scoring across the items in sections A and B. The final score can range from 21-63, where a higher score indicates greater recovery. For greater detail regarding scoring, please see here: https://www.kcl.ac.uk/ioppn/depts/addictions/scales-measures-and-instruments/sure-substance-use-recovery-evaluator

Section C is not scored, but allows people to think through their own priorities in recovery.

Evaluation

Do you think that participants at your RCC would show progress on this measure (i.e., their score would improve over time)?

◯ Yes
◯ No

Please explain: ______________________________

Do you think this measure would be useful to demonstrate the impact of your RCC?

◯ Yes
◯ No

Please explain: ______________________________

Suggested Outcome Measure #4 of 7: Substance Use Recovery Evaluator (SURE)

This scale is a newly developed scale that is a patient-reported outcome measure for assessing recovery from drug and alcohol dependency. It was developed with people in recovery in the United Kingdom. Dr. Joanne Neale recently presented on this scale as part of our seminar series:

https://www.recoveryanswers.org/addiction-research-summaries/seminar-series/

In order to use this scale to assess the impact of your RCC, you would ask participants of your RCC to complete the following questions when they first come to your RCC, and at a later timepoint.

Section A

Thinking about the last week, please rate yourself in each of the following statements.

Drinking and Drug Use (Part 1)

	Never	On 1 or 2 days	On 3 or 4 days	On 5 or 6 days	Every day
I have drunk too much	◯	◯	◯	◯	◯
I have used street drugs	◯	◯	◯	◯	◯
I have experienced cravings	◯	◯	◯	◯	◯

Drinking and Drug Use (Part 2)

	All of the time	Most of the time	A fair amount of the time	A little of the time	None of the time
I have coped with problems without misusing drugs or alcohol	◯	◯	◯	◯	◯
I have managed pains and ill-health without misusing drugs or alcohol	◯	◯	◯	◯	◯
I have been spending my free time on hobbies and interests that do not involve drugs or alcohol	◯	◯	◯	◯	◯

Section B

Still thinking about the last week, please rate yourself in each of the following statements.

Self-Care

	All of the time	Most of the time	A fair amount of the time	A little of the time	None of the time
I have been taking care of my mental health	◯	◯	◯	◯	◯

I have been taking care of my physical health	◯	◯	◯	◯	◯
I have been eating a good diet	◯	◯	◯	◯	◯
I have slept well	◯	◯	◯	◯	◯
I have had a good daily routine	◯	◯	◯	◯	◯

Relationships

	All of the time	Most of the time	A fair amount of the time	A little of the time	None of the time
I have been getting on well with people	◯	◯	◯	◯	◯
I have felt supported by people around me	◯	◯	◯	◯	◯
I have been treated with respect and consideration by people around me	◯	◯	◯	◯	◯
I have treated others with respect and consideration	◯	◯	◯	◯	◯

Material Resources

	All of the time	Most of the time	A fair amount of the time	A little of the time	None of the time
I have had stable housing	◯	◯	◯	◯	◯
I have had a regular income (from benefits, work, or other legal sources)	◯	◯	◯	◯	◯
I have been managing my money well	◯	◯	◯	◯	◯

Outlook on Life

	All of the time	Most of the time	A fair amount of the time	A little of the time	None of the time
I have felt happy with my overall quality of life	◯	◯	◯	◯	◯
I have felt positive	◯	◯	◯	◯	◯
I have had realistic hopes and goals for myself	◯	◯	◯	◯	◯

Section C

Still thinking about the last week, please record how important each of the following have been to you.

	Not important	A little important	Important	Very important
Reducing or abstaining from drinking or drug taking	○	○	○	○
Looking after yourself (physically taking care of yourself, mentally taking care of yourself, having a good diet, sleeping well, having a good routine)	○	○	○	○
Having good relationships with other people (getting on with people, feeling supported by people, being treated with respect, treating others with respect)	○	○	○	○
Having resources and belongings (stable housing, regular income, managing money)	○	○	○	○
Outlook on life (having a good quality of life, feeling positive, having realistic hopes and goals)	○	○	○	○

Scoring

The SURE score is calculated by sum-scoring across the items in sections A and B. The final score can range from 21-63, where a higher score indicates greater recovery. For greater detail regarding scoring, please see here: https://www.kcl.ac.uk/ioppn/depts/addictions/scales-measures-and-instruments/sure-substance-use-recovery-evaluator

Section C is not scored, but allows people to think through their own priorities in recovery.

Evaluation

Do you think that participants at your RCC would show progress on this measure (i.e., their score would improve over time)?

○ Yes
○ No

Please explain: ______________________________

Do you think this measure would be useful to demonstrate the impact of your RCC?

○ Yes
○ No

Please explain: ______________________________

Suggested Outcome Measure #6 of 7: Client Satisfaction Questionnaire

Another way to think about the impact of RCCs is the degree to which the people who come to RCCs are satisfied with the support they receive.

A frequently used measure to assess the satisfaction with services is the "Client Satisfaction Scale". This measure is short (8-items), and can only be used after paying a licensing fee. It was developed a rather long time ago (1979), but has been used a lot since then (in other 2,300 scientific papers).

Larsen DL, Attkisson CC, Hargreaves WA, Nguyen TD. Assessment of client/patient satisfaction: development of a general scale. Eval Program Plann. 1979;2(3):197-207.

In order to use this scale, you would:

- first, need to define what you mean by "services"
- then, ask your RCC participants to answer the following questions in regards to these services:

Client Satisfaction Questionnaire

Note: When we say "service" below, we are referring to the the recovery support the RCC you attend has provided you with.

	Excellent	Good	Fair	Poor
How would you rate the quality of the service you received?	○	○	○	○
	No, definitely not	No, not really	Yes, generally	Yes, definitely
Did you get the kind of service you wanted?	○	○	○	○
	Almost all of my needs have been met	Most of my needs have been met	Only a few of my needs have been met	None of my needs have been met
To what extent has your RCC's services met your needs?	○	○	○	○
	No, definitely not	No, I don't think so	Yes, I think so	Yes, definitely
If a friend were in need of similar help, would you recommend the service to him or her?	○	○	○	○
	Quite dissatisfied	Indifferent or mildly dissatisfied	Mostly satisfied	Very satisfied
How satisfied are you with the amount of help you received?	○	○	○	○
	Yes, they helped a great deal	Yes, they helped somewhat	No, they didn't really help	No, they seemed to make things worse

Have the services you received helped you to deal more effectively with your problems?	○	○	○	○

	Very satisfied	Mostly satisfied	Indifferent of mildly dissatisfied	Quite dissatisfied
In an overall, general sense, how satisfied are you with the service you received?	○	○	○	○

	No, definitely not	No, I don't think so	Yes, I think so	Yes, definitely
If you were to seek help again, would you come back to this service?	○	○	○	○

Scoring

Scores are calculated by summing across items. Items 2, 4, 5, and 8 are reverse scored. Total scores range from 8 to 32, with the higher number indicating greater satisfaction with the services your RCC provided.

Unlike some of the other measures we introduced during this survey, you would NOT ask participants to fill out this scale when they first walk through your doors. Rather, you would ask your RCC participants as some later point after they had a chance to experience your RCC.

Evaluation

Do you think this measure would be useful to demonstrate the impact of your RCC?

○ Yes
○ No

Please explain: ______________________________

You missed a question. If you meant to leave a question blank, please press submit to continue.

Suggested Outcome Measure #7 of 7: Ways in Which RCCs Confer Benefit

Finally, you might want to take a more descriptive and exploratory approach in measuring the impact of your RCC.

Many of the measures we suggested here are psychometrically sound measures, meaning that scientists have spent a lot of time developing and testing each item according to statistical principles.

What we have below is a list of items we made up that we think might capture the many different ways in which RCCs may support people in recovery during their recovery journey.

To use such a descriptive list, you would ask participants at your RCC to rate the following items:

The next questions are about your experience with your RCC. As you are evaluating these items, keep in mind all the services and elements of an RCC, including staff, resources, other participants, etc.

The RCC I attend...

	Strongly disagree	Disagree	Neither agree nor disagree	Agree	Strongly Agree
... gave me confidence to persist in my recovery.	○	○	○	○	○
... was a safe haven in a moment of crisis.	○	○	○	○	○
... gave me trusted advice whenever I needed it.	○	○	○	○	○
... has shown me that recovery is something I can achieve.	○	○	○	○	○
... has shown me that recovery is worth striving for.	○	○	○	○	○
... helped me feel supported in my recovery.	○	○	○	○	○
... gave me a safe space to work through challenges I encounter in my recovery.	○	○	○	○	○
... lets me feel part of a community that accepts me the way I am.	○	○	○	○	○
... has given me access to concrete resources I need to succeed in my recovery.	○	○	○	○	○
... has helped me navigate systems and services that can help me succeed in my recovery.	○	○	○	○	○
... helped me become more at peace with myself.	○	○	○	○	○

... has energized me to move forward with my life, in a way that feels good.	◯	◯	◯	◯	◯
... has provided me opportunities to give back to the community.	◯	◯	◯	◯	◯
... has given me a vision of how to navigate my recovery.	◯	◯	◯	◯	◯
... has taught me concrete steps I can take to progress with my recovery.	◯	◯	◯	◯	◯
... has added joy and friendship to my life.	◯	◯	◯	◯	◯
... keeps encouraging me to stay on track with my recovery.	◯	◯	◯	◯	◯

To report the impact of your RCC in this way, you would simply provide summary statistics for each of the items you assess. For example, you would calculate the percentage of your RCC participants who say "agree" or "strongly agree" for each item.

What other items would you want to add to the list above? In what other ways do RCCs help?

____

Evaluation

Do you think this measure would be useful to demonstrate the impact of your RCC?

◯ Yes
◯ No

Please explain:

____

You missed a question. If you meant to leave a question blank, please press submit to continue.
